# Predicting bacteria causing acute bacterial rhinosinusitis by clinical features^☆^

**DOI:** 10.1016/j.bjorl.2018.12.002

**Published:** 2018-12-29

**Authors:** Dussawan Suwannawong, Kachorn Seresirikachorn, Songklot Aeumjaturapat, Supinda Chusakul, Jesada Kanjanaumporn, Wirach Chitsuthipakorn, Winyu Ruksakul, Kornkiat Snidvongs

**Affiliations:** a Chulalongkorn University, Faculty of Medicine, Department of Otolaryngology, Bangkok, Thailand; b King Chulalongkorn Memorial Hospital, Endoscopic Nasal and Sinus Surgery Excellence Center, Bangkok, Thailand; c Sawan Pracharak Hospital, Department of Otolaryngology, Nakhon Sawan, Thailand; d Nakhon Pathom Hospital, Department of Otolaryngology, Nakhon Pathom, Thailand

**Keywords:** Bacteria, Sinusitis, *Streptococcus pneumoniae*, *Haemophilus influenza*, C-reactive protein, Bactérias, Sinusite, *Streptococcus pneumoniae*, *Haemophilus influenza*, Proteína C-reativa

## Abstract

**Introduction:**

Clinicians rely on clinical presentations to select therapeutic agents for acute bacterial rhinosinusitis. *Streptococcus pneumoniae* and *Haemophilus influenzae* are common in acute bacterial rhinosinusitis. Drug resistant *Streptococcus pneumoniae* and *Haemophilus influenzae* require different antibiotics.

**Objective:**

This study aimed to evaluate the associations between clinical features of acute bacterial rhinosinusitis and pathogenic bacteria.

**Methods:**

Sixty-four patients with acute bacterial rhinosinusitis were enrolled. Clinical features including nasal obstruction, discolored discharge, facial pain, smell disturbance, fever and laboratory findings of patients with acute bacterial rhinosinusitis were collected. The bacterial cultures of endoscopic middle meatal swabs were used as a reference.

**Results:**

Serum C-reactive protein level elevation correlated with the bacterial species (*p* = 0.03), by which was increased in 80.0% of Haemophilus *influenzae* rhinosinusitis and 57.1% of Streptococcus *pneumoniae* rhinosinusitis. The elevated C-reactive protein was the significant predictor for Haemophilus *influenzae* rhinosinusitis with the Odds Ratio of 18.06 (95% CI 2.36–138.20). The sensitivity of serum C-reactive protein level elevation for diagnosing Haemophilus *influenzae* rhinosinusitis was 0.80 (95% CI 0.49–0.94).

**Conclusion:**

Elevation of serum C-reactive protein level was associated with and predicted acute bacterial rhinosinusitis caused by Haemophilus *influenzae*.

## Resumo

**Introdução:**

Os médicos se baseiam nas características clínicas para a escolha dos agentes terapêuticos para o tratamento da rinossinusite bacteriana aguda. *Streptococcus pneumoniae* e *Haemophilus influenzae* são agentes comuns na rinossinusite bacteriana aguda. *Streptococcus pneumoniae* e *Haemophilus influenzae* resistentes a antibióticos requerem medicamentos diferentes.

**Objetivo:**

Avaliar as associações entre as características clínicas da rinossinusite bacteriana aguda e bactérias patogênicas.

**Método:**

O estudo incluiu 64 pacientes com rinossinusite bacteriana aguda. Foram coletadas e registradas as características clínicas, inclusive obstrução nasal, secreção com cor alterada, dor facial, distúrbios do olfato, febre e achados laboratoriais de pacientes com rinossinusite bacteriana aguda. As culturas bacterianas obtidas por *swab* endoscópico do meato médio foram usadas como referência.

**Resultados:**

A elevação do nível sérico de proteína C-reativa estava correlacionada com a espécie bacteriana (*p* = 0,03); ela estava aumentada em 80,0% das rinossinusites por *Haemophilus influenzae* e em 57,1% das rinossinusites por *Streptococcus pneumoniae*. A proteína C-reativa elevada foi um significativo fator preditor de rinossinusite por *Haemophilus influenzae*, com razão de probabilidade de 18,06 (IC 95% 2,36-138,20). A sensibilidade da elevação dos níveis séricos de proteína C-reativa para o diagnóstico de rinossinusite por *Haemophilus influenzae* foi de 0,80 (IC 95% 0,49 ± 0,94).

**Conclusão:**

A elevação dos níveis séricos de proteína C-reativa é um preditor de rinossinusite bacteriana aguda causada por *Haemophilus influenzae*.

## Introduction

Antibiotics should not be routinely prescribed for Acute Rhinosinusitis (ARS) because most cases of the ARS are caused by viruses and almost 70% of the patients with Acute Bacterial Rhinosinusitis (ABRS) may spontaneously resolve without antibiotic treatment.^1^ The common responsible pathogens for ABRS are: *Streptococcus pneumoniae* (20%–45%), *Haemophilus influenzae* (20%–43%), *Moraxella catarrhalis* (14%–28%)^2^, ^3^ and *Staphylococcus aureus* (8%–11%).^4^ When antibiotics are indicated, the narrow-spectrum antibiotics targeting the common pathogens should be prescribed. However, the drug resistance mechanisms of the two common pathogens, *S. pneumoniae* and *H. influenzae* are different, requiring different antibiotic treatments. The mechanism of *S. pneumoniae* resistance to β-lactam antibiotics is DNA mutation. It damages homologous transformation at penicillin-binding proteins^5^ which lowers the affinity of penicillin-binding proteins and counteracts the antibiotic's ability of inhibiting bacterial cell walls biosynthesis. This mechanism requires an increased dose of penicillin.^6^ In contrast, the mechanism of *H. influenzae* resistance to penicillin is the ampicillin resistance gene which produces the β-lactamase enzyme that hydrolyses the beta-lactam ring of the antibiotics. Beta-lactamase inhibitors such as clavulanic acid are recommended in this mechanism.^7^, ^8^ Therefore, the randomly chosen regimen may not be appropriate for both drug resistance mechanisms, and which antibiotic should be the first line for treating ABRS is still controversial. There is no consensus among the international guidelines for the first-line drugs recommendation. The Infectious Diseases Society of America recommends amoxicillin-clavulanate,^9^ while The American Academy of Otolaryngologic Head and Neck Surgery Foundation recommends amoxicillin with or without clavulanate.^10^

Although culturing and identifying the bacteria causing ABRS with the sensitivities to antibiotics are useful, they are not practical. Nasal endoscopy and maxillary sinus puncture require specialist care. These procedures are not available in most primary care settings. Sophisticated microbiology studies are expensive and not generally available. Therefore, clinicians rely on the clinical presentations to select the appropriate therapeutic agents. To date, there are no evidence-based data published on the associations between the clinical features of ABRS and pathogenic bacteria. Different bacteria may cause different clinical presentations according to the bacteria characteristics and virulence. Whether the clinical features can predict the pathogenic bacteria is not known. This study aimed to evaluate the associations between the clinical features and pathogenic bacteria of ABRS and to assess clinical features for predicting the two most common pathogenic bacteria: *S. pneumoniae* and *H. influenzae*.

## Methods

This was a cross-sectional study approved by the Institutional Review Board, Faculty of Medicine, Chulalongkorn University (307/60). All participants provided written informed consents before enrolling to the study. This research did not receive any specific grant from funding agencies in the public, commercial, or nonprofit organizations.

### Participants

From June 2015 to September 2016, consecutive patients with ARS, age 18–65 years from one University hospital in Bangkok: King Chulalongkorn Memorial Hospital and two tertiary provincial hospitals, Sawanpracharak Hospital and Nakornpathom Hospital, were enrolled. These patients were the same patients who were prospectively recruited to a previous study^11^ which assessed the sensitivity and specificity of the international guidelines for diagnosing ABRS. The diagnostic criteria of ARS followed the guideline of Adult Rhinosinusitis by the American Academy of Otolaryngology Head and Neck Surgery.^10^ The criteria included purulent nasal drainage accompanied by nasal obstruction, facial pain-pressure-fullness, or both, with less than 4 weeks duration. The middle meatal bacterial culture was collected under endoscopic guide and the serum sample was collected from each patient on the first visit. The exclusion criteria were (1) The microbiology report was negative for bacteria; (2) patient had received antibiotics or anti-inflammatory drugs (systemic steroids, topical steroids, or NSAIDS) during the previous 2 weeks and (3) patient had received anti-fever drugs within 6 h. Patients were diagnosed with ABRS when the microbiology report was positive for any bacteria. The culture was defined positive using either a quantitative report of ≥10^4^ colony-forming units per milliliter, or a semi-quantitative report of heavy growth or numerous.

### Data collection

Clinical features of each participant were collected, including nasal obstruction, discolored discharge (either patient-reported or from nasal endoscopy), facial pain, smell disturbance, fever >38 °C (either patient-reported or measurement), “double sickening” (clinically worsens after initial improvement), persistent symptoms >10 days, serum C Reactive Protein (CRP), Erythrocyte Sedimentation Rate (ESR) and microbiology report.

### Statistical analysis

Statistical analyses were performed using SPSS v. 17.0 (Statistical Package for the Social Sciences, Chicago, IL). All variables including fever, elevated serum CRP, elevated serum ESR were treated as dichotomous variables. Pearson Chi-Square was used to evaluate the associations between clinical features of ABRS and the pathogenic bacteria. Univariate logistic regression and multivariate logistic regression were used to assess the clinical features for predicting the two most common pathogenic bacteria: *S. pneumoniae* and *H. influenzae*. Sensitivity and specificity of any statistically significant predictors were calculated. The *p*-values of less than 0.05 were considered statistically significant.

## Results

There were 88 patients (67% female) with ARS. A total of 64 participants with positive results of bacterial culture from the middle meatus were included in this study. There were 41 female participants (64.1%). The mean age was 43.4 ± 14.8 years. The mean duration was 16.1 ± 13.6 days. Fourteen patients had positive results for multiple bacteria growths; however, the heavy growth of each patient contained only one bacterial species. The bacterial species identified from the heavy growth of each patient was used for further analysis. Bacterial culture was positive for *S. pneumoniae* in 7 patients (10.9%), *H. influenzae* in 10 patients (15.6%), *M. catarrhalis* in 3 patients (4.7%) and *S. aureus* in 6 patients (9.4%).

### Association between clinical features of ABRS and the pathogenic bacteria

Table 1 shows that the elevated serum CRP level significantly associated with the types of pathogen (*χ*^2^ = 10.9, *p* = 0.03). Eighty percent of the patients with *H. influenzae* infection and 57.1% of the patients with *S. pneumoniae* infection had elevated serum CRP levels. Most patients with other bacterial infections did not have elevated serum CRP level (Table 1). There were no associations between the species of pathogenic bacteria and other clinical features including nasal obstruction, discolored discharge, facial pain, smell disturbance, fever, double sickening, persistent symptoms >10 days, and elevated serum ESR.

**Table 1:** Association between type of causative bacteria and clinical features.

Clinical features	*S. pneumoniae*	*H. influenzae*	*M. catarrhalis*	*S. aureus*	Others	Total	Chi-square	*p*-value
*Nasal obstruction*							3.75	NS
Yes, *n* (%)	3 (43)	3 (30)	0 (0)	2 (33)	6 (16)	14		
No, *n* (%)	4 (57)	7 (70)	3 (100)	4 (67)	32 (84)	50		

*Discolored discharge*							1.85	NS
Yes, *n* (%)	6 (86)	9 (90)	2 (67)	4 (67)	31 (82)	52		
No, *n* (%)	1 (14)	1 (10)	1 (33)	2 (33)	7 (18)	12		

*Facial pain*							6.81	NS
Yes, *n* (%)	7 (100)	4 (40)	2 (67)	4 (67)	21 (55)	38		
No, *n* (%)	0 (0)	6 (60)	1 (33)	2 (33)	17 (45)	26		

*Smell disturbance*							4.61	NS
Yes, *n* (%)	4 (57)	7 (70)	2 (67)	5 (83)	19 (50)	37		
No, *n* (%)	3 (43)	3 (30)	1 (33)	1 (17)	19 (50)	27		

*Fever >38* *°C*							1.44	NS
Yes, *n* (%)	0 (0)	1 (10)	0 (0)	0 (0)	2 (5)	3		
No, *n* (%)	7 (100)	9 (90)	3 (100)	6 (100)	36 (95)	61		

*Double sickening*								NS
Yes, *n* (%)	2 (29)	3 (30)	0 (0)	2 (33)	22 (58)	29	6.99	
No, *n* (%)	5 (71)	7 (70)	3 (100)	4 (67)	16 (42)	35		

*Persistent symptoms*							3.55	NS
Yes, *n* (%)	3 (43)	6 (60)	1 (33)	5 (83)	25 (66)	40		
No, *n* (%)	4 (57)	4 (40)	2 (67)	1 (17)	13 (34)	24		

*Elevated serum CRP*							10.9	0.03
Yes, *n* (%)	4 (57)	8 (80)	1 (33)	1 (17)	11 (29)	25		
No, *n* (%)	3 (43)	2 (20)	2 (67)	5 (83)	27 (71)	39		

*Elevated ESR*							3.97	NS
Yes, *n* (%)	4 (57)	6 (60)	3 (100)	2 (33)	24 (63)	39		
No, *n* (%)	3 (43)	4 (40)	0 (0)	4 (67)	14 (37)	25		

*Total*	7	10	3	6	38	64		

CRP, C reactive protein; ESR, erythrocyte sedimentation rate; NS, not significant.

### Clinical features for predicting *S. pneumoniae* and *H. influenzae* ABRS

When univariate logistic regression was conducted to predict *S. pneumoniae* rhinosinusitis, none of the variables was significant. When univariate logistic regression was conducted to predict *H. influenzae* rhinosinusitis, the elevated CRP was the only significant predictor with the Odds Ratio of 8.71 (Confidence Interval 95% – 95% CI 1.67–45.44).

Similar results were found with multivariate logistic regression. None of the variables predicted *S. pneumoniae* rhinosinusitis. The elevated CRP was the only significant predictor for *H. influenzae* rhinosinusitis with the Odds Ratio of 18.06 (95% CI 2.36–138.20). All patients with *S. pneumoniae* rhinosinusitis had facial pain and none of the patients had fever, therefore, the odds ratios and CIs of these two variables were undefined (Table 1, Table 2).

**Table 2:** Clinical features for predicting causative *Streptococcus pneumoniae* and *Haemophilus influenzae.*

	*Streptococcus pneumoniae* (*n* = 7)		*Haemophilus influenzae* (*n* = 10)	
	Odds Ratio (95% CI)	*p*-value	Odds Ratio (95% CI)	*p*-value
Nasal obstruction	0.07 (0.00–1.91)	NS	1.67 (0.06–47.83)	NS
Discolored discharge	1.42 (0.08–26.94)	NS	1.70 (0.12–23.69)	NS
Facial pain	Undefined	NS	0.23 (0.04–1.30)	NS
Smell disturbance	13.00 (0.45–377.47)	NS	0.31 (0.01–8.68)	NS
Fever >38 °C	Undefined	NS	4.72 (0.25–89.63)	NS
Double sickening	0.62 (0.08–4.95)	NS	0.48 (0.08–2.98)	NS
Persistent symptoms	0.30 (0.04–2.36)	NS	3.00 (0.42–21.66)	NS
Elevated CRP	4.32 (0.25–75.57)	NS	18.06 (2.36–138.20)	0.005
Elevated ESR	0.20 (0.01–3.64)	NS	0.46 (0.07–2.98)	NS

CI, confidence interval; CRP, C reactive protein; ESR, erythrocyte sedimentation rate; NS, not significant.

### Sensitivity and specificity of the significant predictor predicting *S. pneumoniae* and *H. influenzae* ABRS

The elevated CRP, the only significant predictor, was analyzed. The sensitivity and the specificity of elevated serum CRP level in predicting *H Influenzae* infection were 0.80 (95% CI 0.49–0.94) and 0.69 (95% CI 0.55–0.79), respectively. The sensitivity and the specificity of elevated serum CRP level in predicting *S pneumoniae* infection were 0.57 (95% CI 0.25–0.84) and 0.63 (95% CI 0.50–0.74), respectively. The sensitivity and the specificity of elevated serum CRP level in predicting the two most common bacterial infections; either *S. pneumoniae* or *H. influenzae* were 0.71 (95% CI 0.47–0.87) and 0.72 (95% CI 0.58–0.83), respectively (Table 3).

**Table 3:** Sensitivity and specificity of elevated serum CRP for predicting *Streptococcus pneumoniae* and/or *Haemophilus influenzae* ABRS.

	Sensitivity	95% CI	Specificity	95% CI
*Streptococcus pneumoniae*	0.57	0.25–0.84	0.63	0.50–0.74
*Haemophilus influenzae*	0.80	0.49–0.94	0.69	0.55–0.79
*Streptococcus pneumoniae* or *Haemophilus influenzae*	0.71	0.47–0.87	0.72	0.58–0.83

CRP, C reactive protein; ABRS, acute bacterial rhinosinusitis; CI, confidence interval.

## Discussion

To the best of our knowledge, this is the first study assessing clinical symptoms, signs and basic laboratory data for predicting the species of bacteria that causes ABRS. The results of this study showed an association between the elevated serum CRP and the pathogenic bacteria. Serum CRP has been generally used as a marker of inflammation. It is synthesized by the liver. The serum CRP is increased in response to inflammation following interleukin-6 secretion by macrophages and T cells.^12^, ^13^ Compared to ESR, the CRP is more sensitive in identifying the increased inflammatory responses.^14^ The ESR may be normal while the CRP is elevated.

This study found that elevated CRP level was significantly associated with *H. influenzae* rhinosinusitis. This is in agreement with a study by Anttila et al.^15^ When the serum CRP level was monitored daily in children with *H. influenzae* Type B meningitis, the CRP level was elevated when the disease was active, relapsed and with neurologic complications. The CRP level was normal in the recovery period. The results of this study showed a significant association between the elevated CRP and the *H. influenzae* only, but not *S. pneumoniae* or other bacteria. Thomas-Rudolph et al.^16^ showed that serum CRP increased the resistance to nasal infection caused by *S. pneumoniae* in an animal study. The animal study by Mold et al.^17^ showed that the mice receiving intravenous CRP were not infected by viable *S. pneumoniae* injection. CRP binds to the phosphocholine determinant of the cell wall C-polysaccharide from *S. pneumoniae* and provides innate defense against pneumococcal infection.

Antibiotics should not be routinely given to patients with ARS because most cases are of viral origin. Based on the results of this study, the elevated serum CRP appears to be useful in clinical practice. Patients with elevated serum CRP should receive antibiotics for treating *H. influenzae* rhinosinusitis. Amoxicillin with β-lactamase inhibitors such as clavulanic acid should be prescribed if the geographic area has high prevalence of drug resistant *H. influenzae*. Patients with normal serum CRP level may receive symptomatic treatment alone. Antibiotics for treating *S. pneumoniae* should be considered after the conservative treatment has failed. High-dose amoxicillin should be prescribed for drug resistant *S. pneumoniae* in the area with high prevalence.

Serum CRP testing is simple and practical. It takes around 1.5 h for the quantitative testing and as fast as 5 min for the semi-quantitative CRP rapid test. In Denmark and Spain, the rate of antibiotic use was significantly decreased among general practitioners who used the CRP rapid test regarding the requirement of antibiotics for treating rhinosinusitis.^18^, ^19^ A randomized controlled trial by Cals et al. studied two groups of patients with rhinosinusitis. Physicians used CRP assistance in antibiotic prescribing for the first group compared to routine care for the second group. The findings showed similar recovery between groups but the rate of antibiotic use was significantly less when the CRP was used to assist the prescribing decisions.^20^

In line with other studies,^10^, ^21^, ^22^, ^23^, ^24^ we found *S. pneumoniae* and *H. influenzae* were the two major bacteria causing acute bacterial rhinosinusitis. However, the percentages of these two bacteria in our series were lower than other published articles. The recent guideline from the American Academy of Otolaryngology-Head and Neck Surgery Foundation^10^ suggested that *S. pneumoniae* was isolated in 20% to 43% of aspirates, and *H. influenzae* in 22% to 35%. The percentages of *M. catarrhalis* (2% to 10%) and *S. aureus* (10%) in this study were similar to the guideline.

This study had a limited sample size. Although 88 patients with ARS were enrolled in this study and 64 patients had positive bacterial cultures, only 7 and 10 patients were positive to *S. pneumoniae* and *H. influenzae*, respectively. These numbers may be too small and may not have sufficient power for the conclusion. The authors analyzed data from the same population that was already enrolled in a previously published study without sample size calculation.

To date, there are still limited data whether anaerobic bacteria plays a significant role in ABRS. The other limitation of this study was that the bacterial culture was performed only in aerobic bacteria, as anaerobic bacteria culturing is not widely available outside major hospitals. Therefore, the patients with ABRS caused by anaerobes were not included in this study.

This study was a cross-sectional multi-center trial. The participants were enrolled from one university hospital and two provincial public hospitals. Further studies on treatment outcomes of using serum CRP for selecting appropriate antibiotics are needed. Well conducted randomized controlled trials between patients with ABRS who receive either amoxicillin or amoxicillin with clavulanate would help illuminate this complex issue. Serum CRP level and how it relates to cure rates and symptom improvement of each group should be assessed.

## Conclusion

Patients with acute bacterial rhinosinusitis may be treated symptomatically or may require antibiotics depending on the virulence of the pathogenic bacteria. When antibiotics are prescribed, the health care provider can benefit from clues for selecting high dose amoxicillin, suitable for drug-resistant *S. pneumoniae* or amoxicillin with clavulanate for β-lactamase producing *H. influenzae.* The result of this study suggested that the elevation of serum CRP levels favored the use of amoxicillin with β-lactamase inhibitors for treating suspected drug-resistant *H. influenzae* infection.

## Conflicts of interest

The authors declare no conflicts of interest.

## References

[1] Young J., De Sutter A., Merenstein D., van Essen G.A., Kaiser L., Varonen H. (2008). Antibiotics for adults with clinically diagnosed acute rhinosinusitis: a meta-analysis of individual patient data. Lancet.

[2] Fokkens W.J., Lund V.J., Mullol J., Bachert C., Alobid I., Baroody F. (2012). European position paper on rhinosinusitis and nasal polyps 2012. Rhinol Suppl.

[3] Benninger M., Brook I., Farrell D.J. (2006). Disease severity in acute bacterial rhinosinusitis is greater in patients infected with *Streptococcus pneumoniae* than in those infected with *Haemophilus influenzae*. Otolaryngol Head Neck Surg.

[4] Payne S.C., Benninger M.S. (2007). *Staphylococcus aureus* is a major pathogen in acute bacterial rhinosinusitis: a meta-analysis. Clin Infect Dis.

[5] Chaguza C., Cornick J.E., Everett D.B. (2015). Mechanisms and impact of genetic recombination in the evolution of *Streptococcus pneumoniae*. Comput Struct Biotechnol J.

[6] Charpentier E., Tuomanen E. (2000). Mechanisms of antibiotic resistance and tolerance in *Streptococcus pneumoniae*. Microbes Infect.

[7] Townsend C.A. (2002). New reactions in clavulanic acid biosynthesis. Curr Opin Chem Biol.

[8] Tristram S., Jacobs M.R., Appelbaum P.C. (2007). Antimicrobial resistance in *Haemophilus influenzae*. Clin Microbiol Rev.

[9] Chow A.W., Benninger M.S., Brook I., Brozek J.L., Goldstein E.J., Hicks L.A. (2012). IDSA clinical practice guideline for acute bacterial rhinosinusitis in children and adults. Clin Infect Dis.

[10] Rosenfeld R.M., Piccirillo J.F., Chandrasekhar S.S., Brook I., Ashok K., Kramper M. (2015). Clinical practice guideline (update): adult sinusitis. Otolaryngol Head Neck Surg.

[11] Seresirikachorn K., Snidvongs K., Chitsuthipakorn W., Ruksakul W., Chusakul S., Kanjanaumporn J. (2018). EPOS2012 has better specificity compared to IDSA2012 for diagnosing acute bacterial rhinosinusitis. Rhinology.

[12] Pepys M.B., Hirschfield G.M. (2003). C-reactive protein: a critical update. J Clin Investig.

[13] Thompson D., Pepys M.B., Wood S.P. (1999). The physiological structure of human C-reactive protein and its complex with phosphocholine. Structure.

[14] Liu S., Ren J., Xia Q., Wu X., Han G., Ren H. (2013). Preliminary case–control study to evaluate diagnostic values of C-reactive protein and erythrocyte sedimentation rate in differentiating active Crohn's disease from intestinal lymphoma, intestinal tuberculosis and Behcet's syndrome. Am J Med Sci.

[15] Anttila M., Peltola H. (1992). Serum C-reactive protein in the course of *Haemophilus influenzae* Type B meningitis. J Infect Dis.

[16] Thomas-Rudolph D., Du Clos T.W., Snapper C.M., Mold C. (2007). C-reactive protein enhances immunity to *Streptococcus pneumoniae* by targeting uptake to Fc gamma R on dendritic cells. J Immunol.

[17] Mold C., Nakayama S., Holzer T.J., Gewurz H., Du Clos T.W. (1981). C-reactive protein is protective against *Streptococcus pneumoniae* infection in mice. J Exp Med.

[18] Bjerrum L., Gahrn-Hansen B., Munck A.P. (2004). C-reactive protein measurement in general practice may lead to lower antibiotic prescribing for sinusitis. Br J Gen Pract.

[19] Llor C., Bjerrum L., Arranz J., Garcia G., Cots J.M., Gonzalez Lopez-Valcarcel B. (2012). C-reactive protein testing in patients with acute rhinosinusitis leads to a reduction in antibiotic use. Fam Pract.

[20] Cals J.W., Schot M.J., de Jong S.A., Dinant G.J., Hopstaken R.M. (2010). Point-of-care C-reactive protein testing and antibiotic prescribing for respiratory tract infections: a randomized controlled trial. Ann Fam Med.

[21] Brook I. (2011). Microbiology of sinusitis. Proc Am Thorac Soc.

[22] Hadley J.A. (2001). The microbiology and management of acute and chronic rhinosinusitis. Curr Infect Dis Rep.

[23] Huang W.H., Fang S.Y. (2004). High prevalence of antibiotic resistance in isolates from the middle meatus of children and adults with acute rhinosinusitis. Am J Rhinol.

[24] Orlandi R.R., Kingdom T.T., Hwang P.H., Smith T.L., Alt J.A., Baroody F.M. (2016). International Consensus Statement on Allergy and Rhinology: Rhinosinusitis. Int Forum Allergy Rhinol.

